# Intraoral scanner-based monitoring of tooth wear in young adults: 36-month results

**DOI:** 10.1007/s00784-024-05740-0

**Published:** 2024-06-01

**Authors:** Maximiliane Amelie Schlenz, Moritz Benedikt Schlenz, Bernd Wöstmann, Anna Sophia Glatt, Carolina Ganss

**Affiliations:** 1 Department of Prosthodontics, Dental Clinic of the Justus Liebig University Giessen, Giessen, Germany; 2 Department of Oral and Maxillofacial Surgery, Dr. Horst Schmidt Clinic Wiesbaden, Wiesbaden, Germany; 3 Department of Conservative and Preventive Dentistry, Dental Clinic of the Justus Liebig University Giessen, Giessen, Germany; 4 Department of Operative Dentistry, Endodontics and Paediatric Dentistry, Section Cariology of Aging, Dental Clinic of the Philipps-University Marburg, Marburg, Germany

**Keywords:** Tooth wear, Erosion, Attrition, Young adults, Intraoral scanner, Monitoring

## Abstract

**Objectives:**

The study continues our longitudinal observation of wear aiming to further monitoring of progression and lesion morphology and to identify relationships with assumed aetiological factors.

**Materials and methods:**

Molars (FDI #36 or #46) of 74 participants (23.8 ± 2.2 years) were scanned (Trios 3, 3Shape) at the third follow-up (T3; observation period 1,111 ± 10 days). Data sets from T3, T2 (24-month follow-up) and T1 (12-month follow-up) were superimposed with baseline in a 3D analysis software (GOM Inspect). Wear was quantified as maximum vertical tissue loss (µm; median, 95% CI) in various occlusal areas (4/5 cusps and 2 ridges). Morphologies were classified into cupping (C), facet (F), and combined cupping-facet (CF). Aetiological factors were assessed with questionnaires.

**Results:**

Wear increased at T3 significantly at low rates in all areas of the occlusal surface (median between 7.0 (4.0;10.5) and 9.5 (6.0;15.0) µm). There was a clear trend for higher loss values in males, but no association with other factors such as nutrition. C and CF showed significantly higher loss values than F. Areas without initial wear developed F first, which either persisted or developed into C and CF.

**Conclusions:**

Wear continued at low rates with C/CF morphology and sex as significant factors. Cupped lesions seem to develop from facets and thus may not be a valid diagnostic criterion for erosive tooth wear.

**Clinical relevance:**

Wear is a cumulative process that apparently follows complex mechanisms that cannot be conceptualized in simplified terms; C and CF may be indicators for higher progression rates.

**Supplementary Information:**

The online version contains supplementary material available at 10.1007/s00784-024-05740-0.

## Introduction

Tooth wear is a phenomenon that affects all people to a greater or lesser extent, and it is part of the aging process [1]. It is one of the physiological phenomena during the period of use of dentition and is a cumulative process that leads to clinically visible loss of tooth structure over a lifetime.

As long as the amount of wear and the rate of loss are reasonably related to age, there is usually no need for treatment. However, tooth wear can also lead to significant tissue loss, resulting in aesthetic and functional limitations and a need for invasive rehabilitation [2]. Therefore, it would be of great interest to find indicators to help identify such cases in a timely manner and to detect tooth wear at an early stage, allowing for minimally invasive treatment.

The causes of tooth wear are manifold and can be differentiated into chemical and physical factors [3] that affect the tooth surface to varying degrees. This results in different types of wear that manifest themselves as facets or cuppings and are commonly classified as attrition, abrasion, or erosion [4]. However, little is known about the incidence, progression rates and dynamics of wear, and, at least for the wear pattern attributed to erosion, there is little evidence from longitudinal observations of the relationships between aetiological factors and prevalence, incidence and progression [5–7].

Tooth wear can be observed clinically already in adolescents and young adults [8], and progression can be diagnosed visually even in relatively short observation periods [9]. Respectively, studies with intraoral scanners (IOSs) have shown that wear rates between 10 and 100 μm per year may occur on the occlusal surfaces of molars [10–13]. However, wear rates show considerable inter-individual variation [13] and do not appear to be linear [12].

Data from our research group have shown similar results over follow-up periods of 12 and 24 months [14, 15]. These studies included young adults who had one lower first molar digitized with an intraoral scanner. This tooth type is one of the most commonly affected by wear at a young age and has the highest incidence and the greatest progression of wear [13, 16–20]. Therefore, we selected this tooth to investigate in detail the wear phenomena in the different areas of the occlusal surface. In different cusp areas, median wear between 26 and 44 μm was detected in the first year [14] and between 3 and 10 μm in the second year [15], which might indicate a longer-term decrease in the wear rate. As in other studies, we found a considerable range of tissue loss values between individuals, but no correlation between the wear rate and the generally assumed aetiological factors. However, the observation period was still relatively short.

Furthermore, as mentioned above, the occlusal surface also shows different shapes of wear and until now, little is known about how these different defects develop. To date, it has been assumed that facets are the results of the effects of antagonistic contact and movement, whereas cup-shaped cusp tip depressions are thought to be more associated with acid effects [21]. This conceptualizes them as independent phenomena. However, we have first indications from our 24-month observations that cuppings on cusps may not develop without a facet being there first [15]. This would perhaps mean that the two phenomena are not separate entities but rather part of the same process. We have therefore not limited the focus to erosive wear, but our interest was to consider wear as such regardless of its etiology.

We have now continued our study for another year. The aim was to continue monitoring wear quantitatively and to investigate whether the rate of wear would continue to decrease. We also investigated whether aetiological factors could be related to wear rates after a longer observation period. Finally, we continued to monitor the defect morphologies to clarify whether facets and cuppings were indeed sequential phenomena, as suggested by our 24-month data.

## Participants, materials & methods

### Inclusion and exclusion criteria

At the beginning of the study in late 2018, all participants had to be between 18 and 25 years old.

The study teeth were the lower first molars (FDI #36 or #46) that could not have dental restorations covering more than one-third of the occlusal surface. In addition, carious lesions on the study teeth resulted in exclusion from the study. The same conditions applied to the mandatory occlusal antagonist, and the study teeth were checked for contact points in advance. Participants with ongoing orthodontic treatment, visible plaque on the study tooth, and serious general illnesses such as bulimia nervosa were not included in this study.

### Clinical examination

The present study was conducted at the Department of Prosthodontics and the Department of Conservative and Preventive Dentistry of the Justus Liebig University Giessen (Germany) in accordance with the ethical guidelines of the Declaration of Helsinki. The clinical trial was approved by the local ethics committee of the medical faculty of the JLU (ref. no. 148/18) and registered in the German Clinical Trials Register (DRKS00021279). Participants were informed of the procedure and background of the study, and signed an informed consent form. However, they did not receive any specific information about etiological factors for wear or its prevention.

**Fig. 1:**
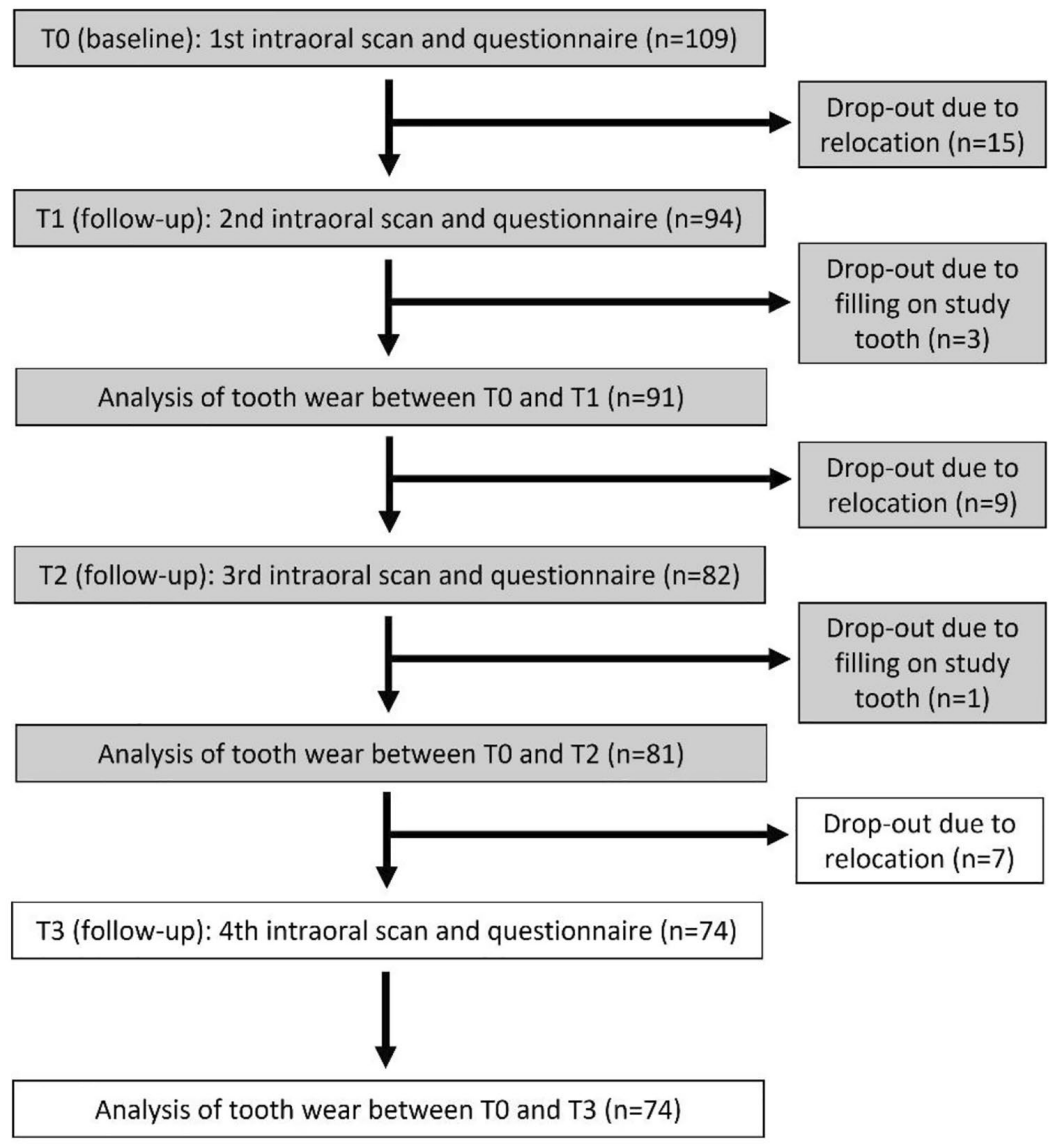
Flow scheme of the clinical study

The present study continues the monitoring of tooth wear and follows up on the data from Schlenz et al. [14, 15]. For this purpose, the data from T0 (baseline) and the data after 12 and 24 months of observation were taken over and are now extended by the 36-month data. A flow scheme of the investigation is displayed in Fig. 1.

Data from 36-month recalls was conducted from November 2018 to December 2022. While a total of 68 female and 41 male dental students with a mean age of 21.0 ± 2.2 years were examined at baseline (T0), due to relocation, 15 subjects dropped out of the study at T1, nine at T2 and seven at T3. In addition, 4 subjects had to be excluded from the study because of restorative treatments performed in the meantime (Fig. 1). For the T3 cohort (*n* = 74), the mean observation period was 369 ± 19 days between T0 and T1, 747 ± 9 days between T0 and T2, and 1,111 ± 10 days between T0 and T3 with 46 females and 28 males (mean age at T3 23.8 ± 2.2 years).

At each time point, intraoral scans (IOS) were taken with the Trios 3 (3Shape, Copenhagen, Denmark), powder-free, using the principle of confocal microscopy. Before this, the IOS was calibrated according to the manufacturer’s instructions, and the specified warm-up time was waited [22]. During the digital impression, which was taken as briefly as possible and strictly according to the scan path recommended by the manufacturer and Müller et al. [23]. The examination lamp of the dental unit was switched off [23, 24]. Relative drying before and during the intraoral scan was performed with dry tips (Microbrush International, Grafton, USA). In addition, the tooth surfaces were dried with an air blower. All subjects were required to brush their teeth before the intraoral scanning.

In the internal software of the intraoral scanner, each subject was assigned a patient case with an anonymized case number, which was used in all study documentation to comply with data protection guidelines. The intraoral scans were stored in Standard Tessellation Language (STL) format, which contains only the three-dimensional data of the digital impressions.

### Superimposition and measurement of tooth wear

For superimposition and measurement of tooth wear, the intraoral scan datasets were imported in STL format into the external 3D analysis software GOM Inspect (version V8 SR1, GOM, Braunschweig, Germany) and stored under the corresponding case number. All procedures and analyses described below were performed by one experienced investigator (A.S.G.) for all subjects.

The scans at time point T0 were cropped to the occlusal surfaces to be examined and defined as target data. The T0 scans were used as a guide when cropping the T1, T2 and T3 scans to ensure that the most comparable scans were available before superimposition. The actual data generated in this way were then pre-aligned to the target using a best-fit algorithm. Following the baseline pre-alignment, the average overlay error was determined. A new best-fit overlay was performed, excluding any areas with an overlay error greater than the average. This iterative process was repeated until the overlay error could no longer be reduced [25]. An average overlay error of ≤ 10 μm was targeted. This procedure was validated in vitro showing that tissue loss after consecutive etching with 35% phosphoric acid, which resulted in loss values of around 10 μm after each exposure, can be reliably measured [26].

**Fig. 2:**
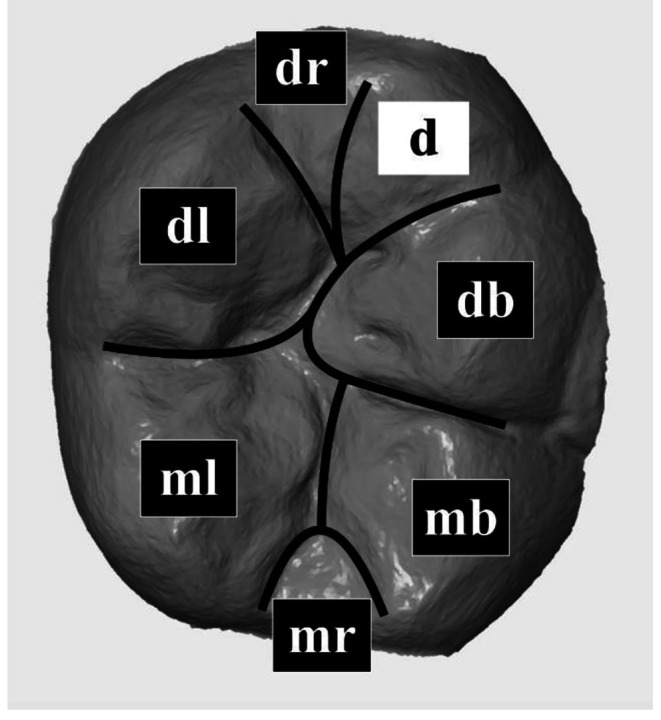
Distribution of the occlusal surface of each study tooth into seven areas: mesiobuccal (mb), distobuccal (db), mesiolingual (ml), distolingual (dl), and if existing distal (d) cusp as well as mesial (mr) and distal ridge (dr)

Upon completion of the generated surface comparison, all examined tooth surfaces were divided into seven areas: mesiobuccal (mb), distobuccal (db), mesiolingual (ml), distolingual (dl) and, if present, distal (d) cusps, as well as mesial (mr) and distal marginal ridge (dr, Fig. 2). Wear was quantified in microns and defined as the maximum vertical distance of the superimposed surfaces in an area of interest. In addition, different lesion morphologies were distinguished, such as cupping (C), facet (F), and combined cupping-facet (CF).

### Questionnaire

In addition to the intraoral scanning, all participants were asked to complete a questionnaire which had been used also at T0, T1 and T2. It assessed several factors related to tooth wear, including dietary habits, frequency of consumption of acidic foods and beverages, taste preferences, chewing habits, presence of heartburn, and use of night guards as described earlier [14]. The questionnaire is available in the supplement.

### Statistics

Statistical analyses were performed with IBM SPSS Statistics version 27 (IBM Germany GmbH, Ehningen, Germany) As there were significant deviations from the Gaussian distribution (Kolmogorov-Smirnov test) non-parametric test procedures (Wilcoxon rank sign, Kruskal-Wallis-Test, Mann-Whitney-Test, Spearman`s rho) were applied. Correspondingly, values for tissue loss as well as acid impacts are given as median and 95% confidence intervals obtained by bootstrapping (method of sampling: simple, number of samples: 1000). The morphology of wear at T0, T1, T2 and T3 is described descriptively. As in the previous studies [14, 15], the mesiobuccal cusp was defined as specific region of interest, and the loss values of this region were used to determine relationships with aetiological factors. Most parameters (sex, wearing a guard, chewing gum, preference for acidic foods and drinks, morphology of the lesions) were expressed as categorical variables; acid impacts were calculated from the individual items of the dietary questionnaires. Details were described earlier [14]. The level of significance was set to < 0.05.

## Results

Table 1 shows the frequencies of wear morphologies in the different areas at T0 and their transformation after 12, 24 and 36 months.

**Table 1:** Cross-tabulation of frequencies of wear morphologies (none, facet, cupping, combined cupping-facet) at baseline (T0) and after 12-month (T1), 24-month (T2) and 36-month (T3) months of monitoring for the different cusp areas (mesiobuccal (mb), distobuccal (db), mesiolingual (ml), distolingual (dl))

		None	Facet	Cupping	Combined cupping-facet	Total (T0)
mb (*n* = 74)						
None T0	T1	5 (100%)				5
	T2	1 (20%)	4 (80%)			
	T3		5 (100%)			
Facet T0	T1		39 (100%)			39
	T2		37 (95%)		2 (5%)	
	T3		36 (92%)		3 (8%)	
Cupping T0	T1			12 (92%)	1 (8%)	13
	T2		1 (8%)	9 (69%)	3 (23%)	
	T3			8 (92%)	5 (8%)	
Combined cupping-facet T0	T1			1 (6%)	16 (94%)	17
	T2				17 (100%)	
	T3				17 (100%)	
db (*n* = 74)						
None T0	T1	8 (80%)	2 (20%)			10
	T2	3 (30%)	7 (70%)			
	T3	2 (20%)	8 (80%)			
Facet T0	T1	1 (2%)	56 (96%)		1 (2%)	58
	T2		56 (96%)		2 (4%)	
	T3		55 (95%)		3 (5%)	
Combined cupping-facet T0	T1				6 (100%)	6
	T2				6 (100%)	
	T3				6 (100%)	
d (*n* = 59)						
None T0	T1	4 (66%)	1 (17%)		1 (17%)	6
	T2	4 (66%)	1 (17%)		1 (17%)	
	T3	3 (50%)	2 (33%)		1 (17%)	
Facet T0	T1		47 (100%)			47
	T2		47 (100%)			
	T3		47 (100%)			
Cupping T0	T1			3 (100%)		3
	T2			3 (100%)		
	T3			3 (100%)		
Combined cupping-facet T0	T1				3 (100%)	3
	T2				3 (100%)	
	T3		1 (33%)		2 (67%)	
ml (*n* = 74)						
None T0	T1	26 (87%)	4 (13%)			30
	T2	19 (64%)	10 (33%)		1 (3%)	
	T3	9 (30%)	19 (63%)		2 (7%)	
Facet T0	T1		39 (100%)			39
	T2		39 (100%)			
	T3		38 (97%)		1 (3%)	
Cupping T0	T1		1 (3%)	2 (67%)		3
	T2		1 (3%)	2 (67%)		
	T3		1 (3%)	2 (67%)		
Combined cupping-facet T0	T1				2 (100%)	2
	T2				2 (100%)	
	T3				2 (100%)	
dl (*n* = 74)						
None T0	T1	20 (95%)	1 (5%)			21
	T2	13 (62%)	8 (38%)			
	T3	6 (29%)	15 (71%)			
Facet T0	T1		47 (100%)			47
	T2		47 (100%)			
	T3		47 (100%)			
Cupping T0	T1		2 (100%)			2
	T2		2 (100%)			
	T3		2 (100%)			
Combined cupping-facet T0	T1				4 (100%)	4
	T2				4 (100%)	
	T3				4 (100%)	

**Fig. 3:**
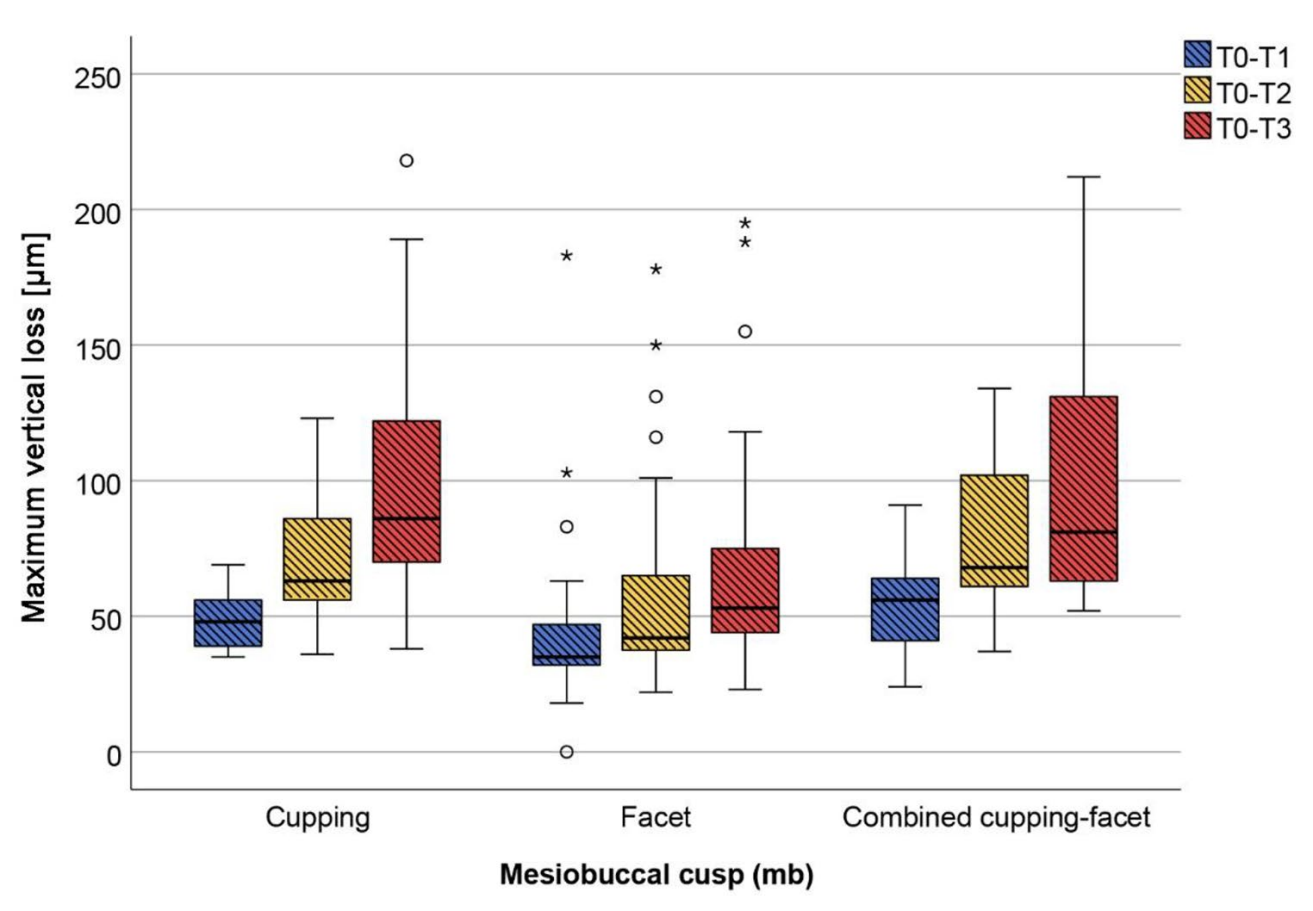
Loss values for the mesiobuccal cusp from the 12-month (blue), 24-month (yellow) to 36-month (red) observational period depending on the wear morphology; circles: outliners; squares: extreme values

**Fig. 4:**
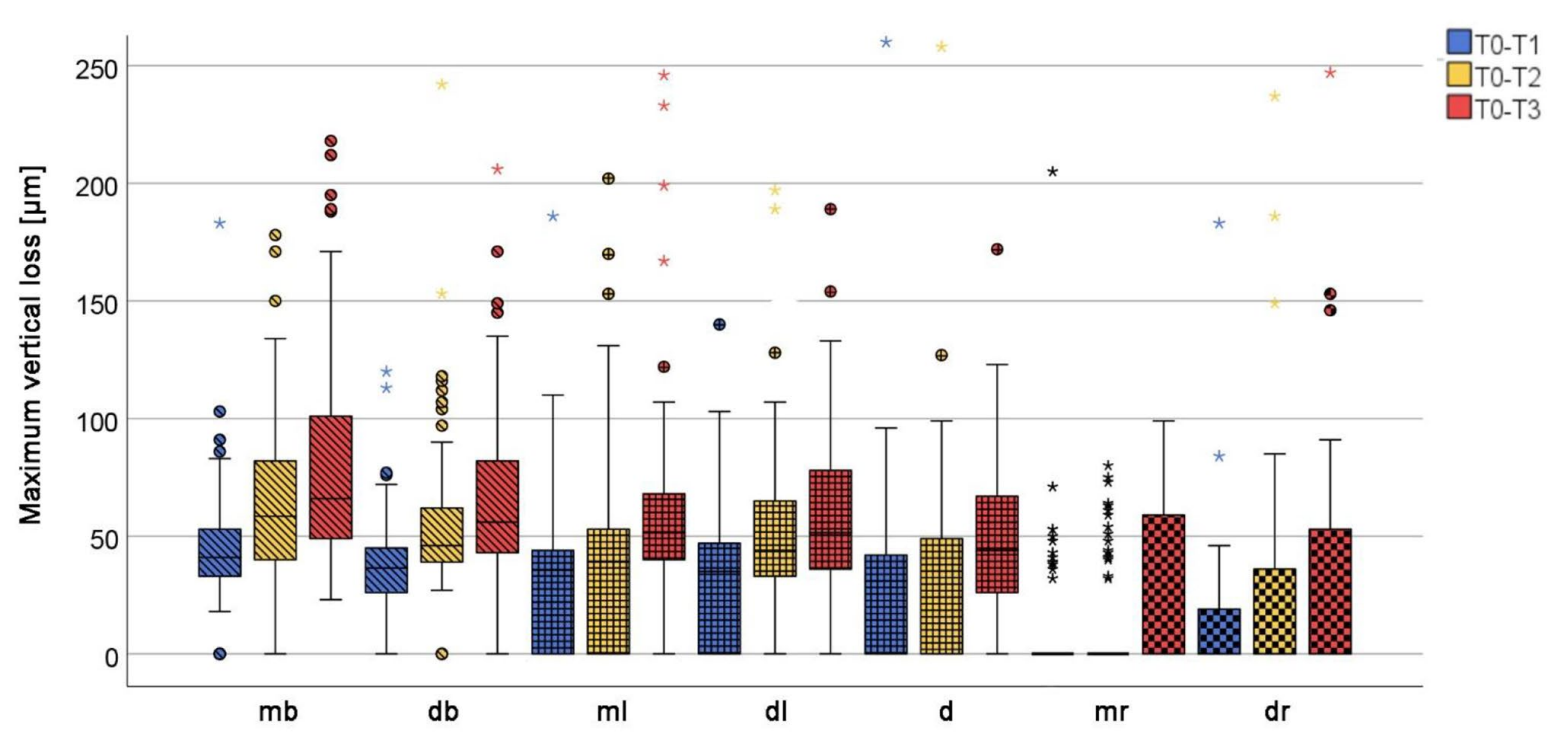
Boxplot diagram of the maximum vertical loss values [µm] for *n* = 74 study teeth after the 12-month (blue), 24-month (yellow) to 36-month (red) observational period distributed to the seven areas (mesiobuccal (mb), distobuccal (db), mesiolingual (ml), distolingual (dl) and if existing distal (d) cusp as well as mesial (mr) and distal ridge (dr)); circles: outliners; squares: extreme values; data cut at 250 μm; p-values for the increase in loss values from one observation time to the next for all areas < 0.01

The mb cusps generally exhibited the greatest variety of lesion morphologies and at T3 no case was not affected by macroscopically visible wear (Fig. 3). It was apparent that the lesion shapes changed slowly over time. On mb cusps that did not show any wear at T0, facets developed first. On those that already had facets, facets continued to be prevalent in most cases, but some developed into combined cupping-facets. A similar picture was seen at the db cusps and to some extent in the d cusps. The three load-bearing cusps mb, db and d wore out in descending order (Fig. 4; Table 2) and this was also reflected in changes in defect morphologies described above, mb being the most pronounced and d the least.

**Table 2:** Annual wear rates (median loss values in µm with 95% CI) for the 12-month (T0-T1), 24-month (T1-T2) and 36-month (T2-T3) observation year (mesiobuccal (mb), distobuccal (db), mesiolingual (ml), distolingual (dl) and if existing distal (d) cusp as well as mesial (mr) and distal ridge (dr); mr-F and dr-F = only marginal ridges with existing facets or facets that were incident within the observation period). Asterisks (** = *p* < 0.001; * = *p* < 0.05) within a row denote significant differences in the loss values compared to the wear rate in the period before (not calculated for marginal ridges)

	T0-T1	T1-T2	T2-T3
mb	42.0 (37.0; 46.5)	15.5 (11.0; 19.00)**	9.5 (6.0; 15.0)
ml	35.5 (23.0; 42.0)	5.5 (0.0; 10.0)**	9.0 (5.0; 15.0)
db	36.5 (34.0; 41.0)	13.0 (8.0; 18.0)**	9.0 (3.0; 13.0)*
dl	35.0 (27.5; 39.0)	9.0 (4.5; 15.5)**	7.0 (4.0; 10.5)
d	31.0 (28.0; 34.0)	3.5 (0,0; 10.0)**	7.0 (4.0;10.5)*
mr	0 (0.0; 0.0)	0 (0.0; 0.0)	0.0 (0.0; 8.0)
mr-F	42.0 (38.5; 51.5) *n* = 12	17.5 (5.0; 50.0) *n* = 18	40.0 (28.0; 55.0) *n* = 35
dr	0 (0.0; 0.0)	0 (0.0; 0.0)	0 (0.0; 0.0)
dr-F	38.0 (35.0; 42.0) *n* = 19	17.0 (3.0; 39.0) *n* = 22	17.0 (6.0; 23.0) *n* = 29

The non-load-bearing lingual cusps also showed increasing wear from T0 to T3, but facets dominated here. At least in ml cusps, however, the path from no wear through facets to cuppings or combined cupping-facets was also emerging.

The marginal ridges also showed macroscopic wear, which, however, appeared exclusively as facets. At T0, 84% of the mesial marginal ridges had no wear; of these, 5% had facets at T1, 14% at T2 and 40% at T3. Of the distal marginal ridges, 74% had no wear at T0; of these 7% had developed facets at T1, 14% at T2 and 27% at T3. Four cases showed extremely high loss values (> 900 μm), which were visible as macrofractures on the scans.

In the cusp areas, the cumulative loss values continued to increase significantly from year to year (*p* < 0.01 each; Fig. 4) but the annual wear rate changed (Table 2). Compared to the first year, the rate of progression decreased significantly in the second year and continued in the third year at a similarly low level. Overall, a considerable interindividual range of wear was observed, but the individual annual wear rates were not significantly correlated. At the marginal ridges, wear occurred only in areas where facets were present or incident whereas no wear was quantified for marginal ridges that did not show macroscopic wear (Table 2).

When looking at the factors that may be associated with loss (Table 3), only sex and wear morphology were identified as significant. The most consistent relationship was found between wear morphology and loss. Cuppings or combined cupping-facets always exhibited higher loss values than facets, both prospectively from T0 (except loss in the third year, where a respective difference did not reach significance) and retrospectively from T3.

**Table 3:** Relationship of loss values (µm), wear morphologies and assumed aetiological factors (numerical values: median with 95% CI); cupping (C), facet (F), combined cupping-facet (CF).

Item/con-dition	Category				*p*-value
Sex		Male *n* = 28	Female *n* = 46		
	Loss T0-T1	48.5 (38.5; 56.59	38.5 (35.0;45.0)		0.052
	Loss T0-T2	68.0 (60.5; 82.5)	46.5 (42.0; 60.5)		**0.023**
	Loss T0-T3	82.0 (75.5; 107.5)	63.5 (53.0; 45.0)		0.125
	Loss T1-T2	25.0 (16.0; 30.0)	12.5 (6.5; 16.5)		**0.026**
	Loss T2-T3	7.5 (3.0; 27.0)	10.0 (6.0; 16.0)		0.053
Night guard		No *n* = 40	Yes *n* = 34		
	Loss T0-T3	64.5 (53.0; 84.0)	66.5 (53.0; 76.0)		0.849
Chewing gum		No *n* = 47	Yes *n* = 27		
	Loss T0-T3	75.0 (66.0; 94.0)	62.0 (50.0; 70.0)		0.132
I like acidic food		No *n* = 28	Neither *n* = 12	Yes *n* = 36	
	Loss T0-T3	60.0 (48.0; 93.5)	72.5 (50.5; 92.0)	66.0 (53.0; 73.0)	0.988
I like acidic drinks		No *n* = 45	Neither *n* = 10	Yes *n* = 19	
		62.0 (51.0; 76.0)	68.5 (49.0; 94.0)	70.0 (53.0; 98.0)	0.812
Morphology at T0		F *n* = 39	C/CF *n* = 30		
	Loss T0-T3	53.0 (48.0;66.0)	83.5 (68.5; 111.5)		**< 0.001**
	Loss T1	35.0 (33.0; 43.0)	50.0 (45.5; 58.5)		**< 0.001**
	Loss T2	11.0 (5.0; 15.0)	19.5 (14.0; 28.0)		**0.035**
	Loss T3	8.0 (5.0; 15.0)	13.5 (5.5; 27.0)		0.145
Morphology at T3		F *n* = 41	C/CF *n* = 33		
	Loss T0-T3	51.0 (46.0;62.0)	86.0 (74.0; 118.0)		**< 0.001**
	Loss T1	35.0 (32.0; 43.0)	49.0 (45.0; 57.0)		**< 0.001**
	Loss T2	12.0 (5.0;16.0)	20.0 (17.0; 28.0)		**0.049**
	Loss T3	7.0 (2.0; 10.0)	20.0 (10.0; 28.0)		**0.019**
	Acid impacts T0	2.2 (1.7; 2.5)	2.1 (1.5; 4.0)		0.515
	Acid impacts T1	2.9 (2.2; 3.0)	2.7 (1.9; 3.7)		0.909
	Acid impacts T2	2.5 (1.9; 2.9)	2.7 (1.9; 3.6)		0.617
	Acid impacts T3	2.2 (1.7; 2.9)	2.5 (1.6; 3.5)		0.293
	Mean acid impacts T0-T3	2.3 (1.8; 2.9)	2.8 (2.3; 3.5)		0.399

The relationship between loss and sex was inconsistent; females had generally numerically lower loss values than males except for the last year of observation, but these differences did not always reach significance. All other factors (wearing a night guard, frequency of chewing gum, preferences for acidic foods and drinks, and acid impacts during observation periods) showed no relationship to the loss values. Also, at no time during the entire observation time did subjects with cuppings or combined cupping-facets on the mb cusps have more acid impulses than those with facets (Table 3). There was no significant association between tissue loss values and acid impacts (loss T0 to T3 and mean acid impacts T0 to T3: *r* = 0.084; *p* = 0.474; loss T2 to T3 and acid impacts T2 to T3: *r* = 0.196; *p* = 0.097).

**Fig. 5:**
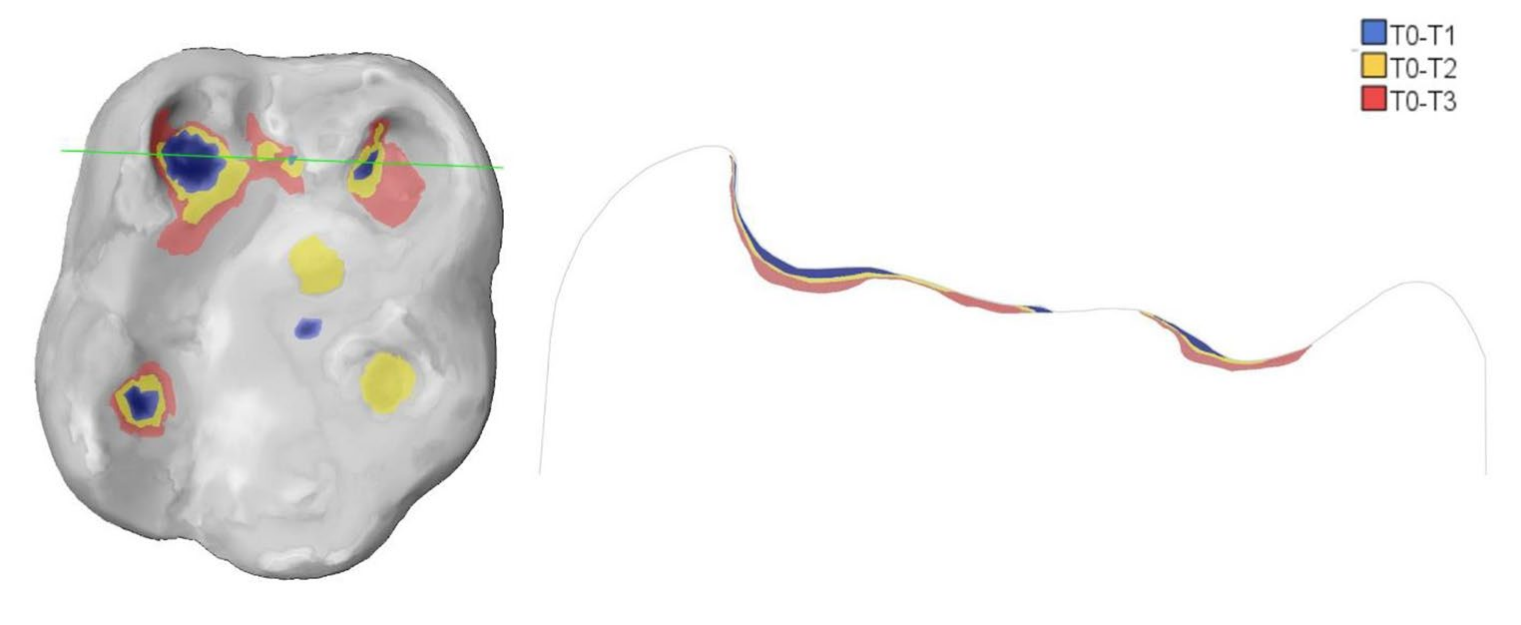
Example of study tooth #46 with different colored areas marking the evolution of tooth wear from Baseline (grey), 12-month (blue), 24-month (yellow) to 36-month (red) as occlusal view (left) and in cross-section (right). This tooth already presented a considerable tissue loss with cuppings at the beginning, these deepened in the further course. However, new lesions also developed, such as the cupping lesion of the distobuccal cusps at 24-month (yellow)

**Fig. 6:**
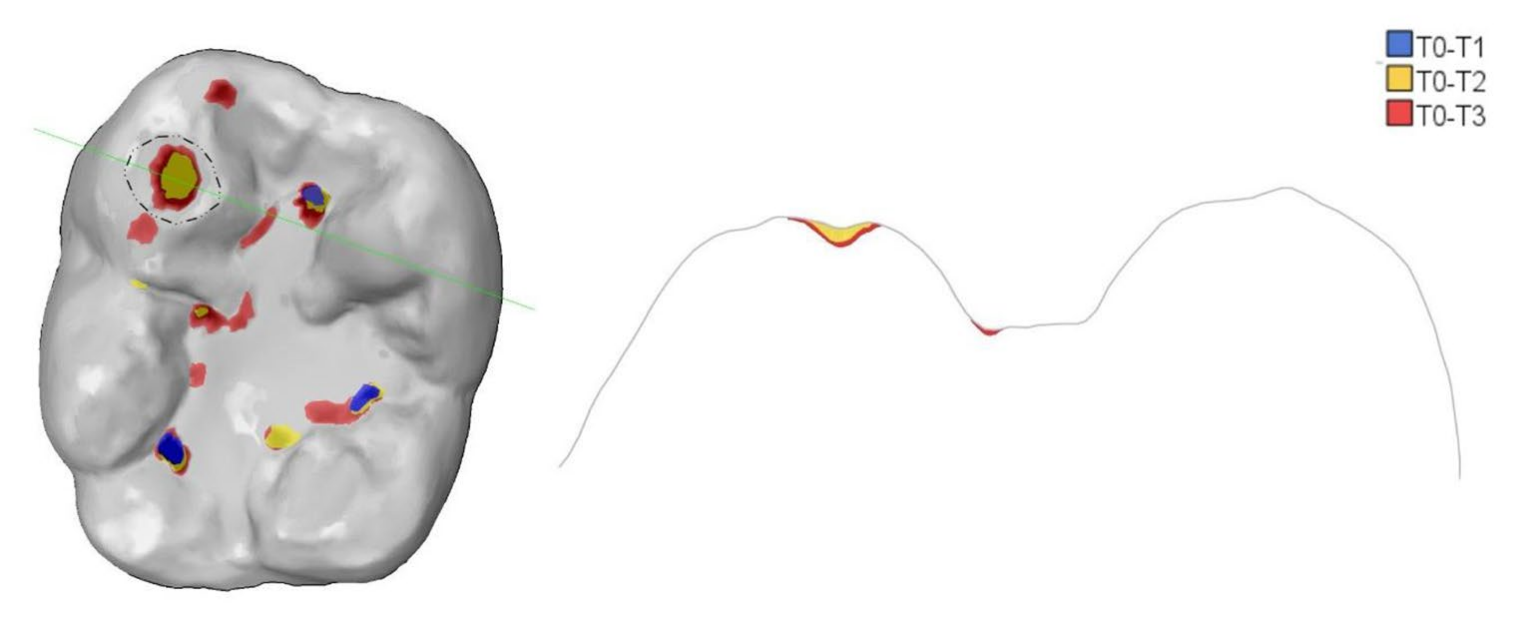
Example of study tooth #36 with different coloured areas marking the evolution of tooth wear from Baseline (grey), 12-month (blue), 24-month (yellow) to 36-month (red) as occlusal view (left) and in cross-section (right). Note that there was no tissue loss in the facet area (dashed circle) in the first year. In the second year, a small cupping (yellow) appeared in the facet area, which had deepened further in the third year (red)

Figures 5 and 6 show an example of study tooth #46 and #36 with different colored areas marking the evolution of tooth wear from T0 to T3.

## Discussion

We were able to continue our observational study on the monitoring of tooth wear into the third year. Of the original 109 participants, we were able to monitor 74 who were still enrolled. Since the dropouts were due to reasons that cannot be linked to the content of the study (most of them moved, the remaining had fillings in their study teeth), we can state that the data are missing completely at random so that we do not expect any bias.

Overall, the loss values of the third year confirm the relatively low wear rates of the second year. However, as in the previous observation periods, significant inter-individual differences were found. Such considerable variations in the amount of wear were also reported in other contexts [10] and were also evident in a group of patients who already had moderate to severe tooth wear [13]. However, wear rates also seem to vary not only inter-individually, but also within an individual, as we could not find a correlation between the individual progression rates in the three observation periods in our study. This means that a high wear rate in one observation period is not an indicator for high wear rates in the future.

The question remains as to how such findings can be explained. One obvious and often investigated factor is exposure to acid from food and drink, but also from stomach contents. The latter seems to have played a minor role here, as symptoms of reflux or frequent vomiting were not reported in our group of subjects. However, we enquired in great detail about exposure to acids from food and drink and attempted to associate this in a subtle way with the observed wear rates. Similar to the two previous observation periods, however, we found no association between these exogenous acid exposures and tissue loss.

It remains to be assumed that there must be as yet unknown factors that influence the wear process more than what has been suggested so far, or that many factors interact in such a complex way that the role of individual factors remains concealed. For example, dietary factors with many acid impacts or a high proportion of coarse food may only lead to increased wear in combination with genetic factors that influence the acid solubility and hardness of dental hard tissues; in addition, there could also be simultaneous contributing factors such as saliva [27], chewing force or the action of antagonistic teeth.

Thus, many factors could enhance or alleviate each other, resulting in a complex and perhaps inexplicable interplay. Furthermore, all diagnostic procedures are subject to limitations. Behavioral factors in particular could only be recorded using questionnaires, which may be far too crude an instrument.

However, a consistent observation across all three years was that the morphology of wear lesions plays a role. Therefore, this time we focused in particular on how wear develops morphologically. It is generally assumed that the different forms of wear such as facets or cuppings are based on different aetiologies; at least that different factors are at the foreground in the complex mechanisms of wear. For example, facets are assumed to be the result of two-body wear, in which antagonistic tooth surfaces act against each other, while abrasions are described as three-body wear. Here, for example, during the chewing process, the hardness of a bolus determines the wear rates while the microhardness of the antagonistic tooth surfaces relative to each other (i.e. enamel and dentin) determines the defect resulting from the action of the bolus. Thus, the softer dentin is expected to develop cuppings, while the harder enamel wears only slightly. The same was assumed for erosive tooth wear, but here the acid impact reduces the microhardness of the tooth’s hard tissue, which should then lead to increased three-body wear [28, 29]. Accordingly, wear on occlusal surfaces caused by the abrasion of coarse food or by the abrasion of less coarse food after the reduction of microhardness by acids should result in similar defect shapes [30]. Based on such wear mechanisms, the higher wear rate in cuppings and combined cupping-facets, which we have now observed again, would be due to the exposure of the softer dentine, which wears faster than the harder enamel. However, this theory was at least partially refuted, as cuppings could also be produced in enamel without dentine exposure, at least experimentally [31].

So far, however, almost nothing is known about how wear morphologies change over time. We have used two cases to illustrate the evolution of the wear process by superimposing the occlusal surfaces at the four observation points. In one case with more advanced wear and an occlusal surface that had already initially lost its physiological morphology, the wear appears to simply continue. At the different times of examination, some new areas were affected while the wear stagnated in other areas. In the other case, one facet was initially present, but in the first 12 months no progression was detected. Whereas, after 24 months the facet developed to a cupping with progression after 36 months.

It was a very interesting observation that in areas without wear, facets always occurred first, which then continued to grow into a combined cupping-facet. Enamel behaves in a complicated manner under the various two- and three-body wear conditions [32, 33]. For example, in a three-media wear process, not only the hardness of a particle but also its size seems to be important. Such factors can lead to macrofractures, as we have observed in marginal ridges, for example, where dental hard tissues can be lost in the order of millimeters; microfractures can be caused by cracks starting on the surface and may manifest themselves in cupping-like shapes, while microfractures caused by tiny hard particles can lead to small, even wear areas on the surface [33]. Further, several fatigue mechanisms in enamel were described [32].

Suppose we assume that the wear we observed in our group of individuals is mainly physically induced and that microwear first creates a facet that forms cuppings over time via cracking and contact fatigue mechanisms [32]. In that case, it explains the finding that we could not find any relation to dietary factors. We can then perhaps also explain the strong tendency for females to have less tissue loss than males. Females have a lower bite force [34, 35], so lower loads act on the tooth surfaces. If we assume that enamel cracks play a relevant role in the wear process, the healing tendency for such cracks could be important. In this context, it has been shown that enamel from female donors seems to be more effective at healing than that from male donors [36]; if both factors interact, this could at least partly explain the lower tendency to wear in women.

However, all these considerations remain speculative but encourage further investigation.

Our study has several limitations. Firstly, we focused on the occlusal surface of a single tooth and no conclusions can be drawn about wear in other areas of the dentition. This could also be shown in another study with patients with moderate to severe erosive tooth wear. However, for future studies, especially in older age groups, it would be very important to examine also other areas and the full arch. Furthermore, we only measured one loss value per tooth area, and this only provides limited information about the extent of wear in terms of volume: Thus, the superimposition of scans over time indicates, that the area of the loss also plays a role in addition to the maximum value. Other measurement methods may be able to provide further information on wear mechanisms and associated risk factors.

However, a strength of our study is that we observed the morphology of wear lesions longitudinally. As wear was so far a relatively slow process in our group of younger individuals, it was only the longer observation period of three years that allowed us to identify changes in the wear shapes. This opens a new look at the theories on the causes and diagnosis of tooth wear.

## Conclusion

Also after 36 months, we observed a significant increase in wear in all areas of the occlusal surface. The relatively high wear rate after the first year decreased in the second year and continued similarly in the third year. Wear seems to present itself as a facet at first, while cuppings only appear to develop in the later wear process. The frequently assumed aetiological factors showed no relationship to the loss values even after 36 months. From these observations, it can be deduced that cuppings on the cusp tips may not be a valid diagnostic criterion for erosive tooth wear.

### Electronic supplementary material

Below is the link to the electronic supplementary material.

Supplementary Material 1

## Data Availability

No datasets were generated or analysed during the current study.

## References

[1] Bartlett D, O’Toole S (2020). Tooth wear: best evidence consensus statement. J Prosthodont.

[2] Hardan L, Mancino D, Bourgi R, Cuevas-Suarez CE, Lukomska-Szymanska M, Zarow M, Jakubowicz N, Zamarripa-Calderon JE, Kafa L, Etienne O (2022). Treatment of tooth wear using direct or indirect restorations: a systematic review of clinical studies. Bioeng (Basel).

[3] Oudkerk J, Grenade C, Davarpanah A, Vanheusden A, Vandenput S, Mainjot AK (2023). Risk factors of tooth wear in permanent dentition: a scoping review. J Oral Rehabil.

[4] Schlueter N, Amaechi BT, Bartlett D, Buzalaf MAR, Carvalho TS, Ganss C, Hara AT, Huysmans M, Lussi A, Moazzez R (2020). Terminology of erosive tooth wear: Consensus report of a workshop organized by the orca and the cariology research group of the iadr. Caries Res.

[5] Brusius CD, Alves LS, Susin C, Maltz M (2018). Dental erosion among south Brazilian adolescents: a 2.5-year longitudinal study. Community Dent Oral Epidemiol.

[6] El Aidi H, Bronkhorst EM, Huysmans MC, Truin GJ (2011). Multifactorial analysis of factors associated with the incidence and progression of erosive tooth wear. Caries Res.

[7] Marro F, O’Toole S, Bernabe E, Bartlett D, Aranguiz V (2022). Associated risk factors with quantitative erosive tooth wear progression. J Dent.

[8] Schlueter N, Luka B (2018). Erosive tooth wear - a review on global prevalence and on its prevalence in risk groups. Br Dent J.

[9] Marro F, De Lat L, Martens L, Jacquet W, Bottenberg P (2018). Monitoring the progression of erosive tooth wear (etw) using bewe index in casts and their 3d images: a retrospective longitudinal study. J Dent.

[10] Lambrechts P, Braem M, Vuylsteke-Wauters M, Vanherle G (1989). Quantitative in vivo wear of human enamel. J Dent Res.

[11] Leon Velastegui M, Montiel-Company JM, Agustin-Panadero R, Fons-Badal C, Sola-Ruiz MF (2022). Enamel wear of antagonist tooth caused by dental ceramics: systematic review and meta-analysis. J Clin Med.

[12] Esquivel-Upshaw JF, Rose WF, Barrett AA, Oliveira ER, Yang MC, Clark AE, Anusavice KJ (2012). Three years in vivo wear: Core-ceramic, veneers, and enamel antagonists. Dent Mater.

[13] Bronkhorst H, Bronkhorst E, Kalaykova S, Pereira-Cenci T, Huysmans MC, Loomans B (2023). Inter- and intra-variability in tooth wear progression at surface-, tooth- and patient-level over a period of three years: a cohort study.: Inter- and intra-variation in tooth wear progression. J Dent 104693.

[14] Schlenz MA, Schlenz MB, Wöstmann B, Jungert A, Ganss C (2021). Intraoral scanner-based monitoring of tooth wear in young adults: 12-month results. Clin Oral Investig doi.

[15] Schlenz MA, Schlenz MB, Wöstmann B, Glatt AS, Ganss C (2023). Intraoral scanner-based monitoring of tooth wear in young adults: 24-month results. Clin Oral Investig.

[16] El Aidi H, Bronkhorst EM, Truin GJ (2008). A longitudinal study of tooth erosion in adolescents. J Dent Res.

[17] El Aidi H, Bronkhorst EM, Huysmans MC, Truin GJ (2010). Dynamics of tooth erosion in adolescents: a 3-year longitudinal study. J Dent.

[18] Ganss C, Klimek J, Giese K (2001). Dental erosion in children and adolescents–a cross-sectional and longitudinal investigation using study models. Community Dent Oral Epidemiol.

[19] Hasselkvist A, Johansson A, Johansson AK (2016). A 4 year prospective longitudinal study of progression of dental erosion associated to lifestyle in 13–14 year-old Swedish adolescents. J Dent.

[20] Khan F, Young WG, Law V, Priest J, Daley TJ (2001). Cupped lesions of early onset dental erosion in young southeast queensland adults. Aust Dent J.

[21] Ganss C, Lussi A (2014). Diagnosis of erosive tooth wear. Monogr Oral Sci.

[22] Rehmann P, Sichwardt V, Wöstmann B (2017). Intraoral scanning systems: need for maintenance. Int J Prosthodont.

[23] Müller P, Ender A, Joda T, Katsoulis J (2016). Impact of digital intraoral scan strategies on the impression accuracy using the trios pod scanner. Quintessence Int.

[24] Arakida T, Kanazawa M, Iwaki M, Suzuki T, Minakuchi S (2018). Evaluating the influence of ambient light on scanning trueness, precision, and time of intra oral scanner. J Prosthodont Res.

[25] Güth JF, Erdelt K, Keul C, Burian G, Schweiger J, Edelhoff D (2020). In vivo wear of cad-cam composite versus lithium disilicate full coverage first-molar restorations: a pilot study over 2 years. Clin Oral Invest.

[26] Witecy C, Ganss C, Wöstmann B, Schlenz MB, Schlenz MA (2021). Monitoring of erosive tooth wear with intraoral scanners in vitro. Caries Res.

[27] Madariaga VI, -Cenci TP, Walboomers XF, Loomans BAC (2023). Association between salivary characteristics and tooth wear: a systematic review and meta-analysis. J Dent.

[28] Mair LH, Stolarski TA, Vowles RW, Lloyd CH (1996). Wear: mechanisms, manifestations and measurement. Report of a workshop. J Dent.

[29] d’Incau E, Couture C, Maureille B (2012). Human tooth wear in the past and the present: tribological mechanisms, scoring systems, dental and skeletal compensations. Arch Oral Biol.

[30] Ganss C, Klimek J, Borkowski N (2002). Characteristics of tooth wear in relation to different nutritional patterns including contemporary and medieval subjects. Eur J Oral Sci.

[31] Ruben JL, Roeters FJM, Truin GJ, Loomans BAC, Huysmans M (2019). Cup-shaped tooth wear defects: more than erosive challenges?. Caries Res.

[32] Kruzic JJ, Hoffman M, Arsecularatne JA (2023). Fatigue and wear of human tooth enamel: a review. J Mech Behav Biomed Mater.

[33] Lucas PW, van Casteren A (2015). The wear and tear of teeth. Med Prin Pract.

[34] Calderon PS, Kogawa EM, Lauris JR, Conti PC (2006). The influence of gender and bruxism on the human maximum bite force. J Appl Oral Sci.

[35] Shiga H, Komino M, Yokoyama M, Sano M, Arakawa I, Nakajima K, Fujii S (2023). Relationship between age and occlusal force in adults with natural dentition. Odontology.

[36] Rivera C, Arola D, Ossa A (2013). Indentation damage and crack repair in human enamel. J Mech Behav Biomed Mater.

